# How does a move towards a coaching approach impact the delivery of written feedback in undergraduate clinical education?

**DOI:** 10.1007/s10459-021-10066-7

**Published:** 2021-09-14

**Authors:** Amanda Roberts, Mark Jellicoe, Kathryn Fox

**Affiliations:** Education Research and Scholarship Group, The School of Dentistry, The University of Liverpool, Pembroke Place, Liverpool, L3 5PS United Kingdom

**Keywords:** Feedback, Learner educator partnership, Coaching, Mentoring, Self-regulated learning

## Abstract

Feedback uptake relies on interactions between learners and educators Winstone (Educ Psychol 52: 17–37, 2017). Feedback that coaches using a feedforward approach, is considered to be more personal and emotionally literate Bussey (Bull R Coll Surg Engl 99: 180–182, 2017), Hattie (Rev Educ Res 77: 81–112, 2007). Many modes of feedback are employed in clinical teaching environments, however, written feedback is particularly important, as a component of feedback discourse, as significant time may elapse before a similar clinical situation is encountered. In practice, time constraints often result in brief or descriptive written feedback rather than longer coaching feedback. This study aimed to explore whether a change in ethos and staff development would encourage clinical dental tutors to utilise a coaching approach in their written feedback. Across two time-points, written feedback was categorised into either descriptive, evaluative or coaching approaches. Cross-sections of data from 2017 to 2019 were examined to determine whether changes in practice were noted and whether there were any alterations in the affective nature of the language used. Feedback moved significantly towards coaching and away from a descriptive approach. A shift towards the use of more positive language was seen overall, although this was solely driven by a change in the evaluative feedback category. Descriptive feedback generally used neutral language with coaching feedback using marginally more positive language. Both categories employed significantly lower levels of affective language than evaluative feedback. These data indicate a move towards feedback approaches and language that may support increased uptake and utilisation of feedback.

**Supplementary Information:**

The online version contains supplementary material available at 10.1007/s10459-021-10066-7.

## Introduction

Educators use feedback as an intervention to support learner development (Hattie & Timperley, 2007). In support of the development of feedback literacy, practice has recently shifted to encourage proactive recipience (Allen & Molloy, 2017; Noble et al., 2020) and continuous review (Tripodi et al., 2020). Although learners are seen as active agents in this developmental process (Hattie & Timperley, 2007; Winstone et al., 2017a, b), learners and educators may hold different perspectives on feedback (Ozuah et al., 2007). If feedback messages are to translate to action which support the learner in implementing change, educators must be aware of complex interplay of factors, such as interpersonal relationships, delivery of message and emotions. These factors can act as barriers to development if institutional systems are not appropriately designed and supported (Freeman et al., 2020; Jellicoe & Forsythe, 2019; Winstone et al., 2017a, b; Weidinger et al., 2016).

Following their colleagues in other areas of Higher Education (HE), learners in clinical settings report dissatisfaction with feedback, commonly citing inadequate guidance to support improvement (Hesketh & Laidlaw, 2002; Noble et al., 2020). Dawson and colleagues (2019, p. 25) report that effective feedback comments ‘are usable, detailed, considerate of affect and personalised to the student’s own work’. In addition, Freeman et al. (2020) indicated that learners in UK dental schools value feedback when institutional processes are well designed and supported. However, several authors suggest that many clinicians have no training as educators, and therefore are not aware of how to give good feedback, often having been recipients of poor feedback themselves (Bush et al., 2013; Clynes & Raftery, 2008; Tekian et al., 2017). Continuing to support educator understanding of the content and context of feedback messages, that guide learners towards the next stage in their development journey, appears justified (Hattie & Timperley, 2007; Tripodi et al., 2020; Winstone et al., 2017a, b).

In order to improve the usability of feedback, a mentoring, or coaching, role has been described by Bussey and Griffiths (2017) and others (Stone & Heen, 2015). Effective coaching feedback is suggested to create opportunities for self-reflection in learning and supports lifelong learning (Reynolds, 2020). Two alternate, more traditional, but less effective, educator feedback roles are also described by Bussey and Griffiths (2017). First, an administrator approach highlights what has happened during learning, offering no advice. Next, the examiner role introduces a qualitative, subjective evaluation of the quality of the learning but little additional information and therefore fail to offer the ‘where next?’ guidance that learners seek. In addition, the impersonal nature does not foster a learner-educator alliance (Ajjawi & Regehr, 2019; Hattie & Timperley, 2007).

Emotions in feedback are a contested space. For example, the much-used ‘feedback sandwich’ is reported to blunt the emotional impact of feedback for both learner and educator. Despite the popularity of the approach, its utility is reported to be low (Molloy et al., 2020). Low utility feedback practices, can focus the learner on extrinsic motivation, strategic or surface approaches (Alonso-Tapia & Pardo, 2006; Carless & Boud, 2018) and may result in a climate of fear (Fox, 2019). Therefore a challenge for educators in HE is to support learners to negotiate emotional uncertainty as part of the developmental conversation. In this approach learners and educators must be able to evaluate feedback messages, recognising the emotional barriers and strategies that smooth the path to development and self-regulated learning (Molloy et al., 2020; Winstone et al., 2017a, b; Panadero, 2017). Developing understanding surrounding the emotional content of feedback messages, through a coaching approach to feedback, may help to drive changes in feedback practice that will ultimately support learners (Reynolds, 2020; Tripodi et al., 2020) Other barriers that may exist, particularly when learners do not value feedback, or have less effective learning strategies, is feedback accessibility. Learners have reported being overwhelmed with volumes of feedback, particularly when delivered via learning management systems (Winstone et al., 2020). Review evidence indicates differential effects of message length on feedback uptake in medical education domains vary according to task (Ridder et al., 2015). It is not clear how this varies according to feedback typology discussed here. Determining this may support developing understanding in this area.

### The current study

Supporting the need for change, learners in the current educational setting had remarked on the variable quality of in terms of improvement messages they received, the emotional content and length of the comments, leading some learners to report feeling demoralised and suggesting a tendency towards low value, low utility feedback. Therefore, feedback practice did not appear to provide conditions that pre-disposed learners towards engagement (To, 2021). A need for a change of ethos was therefore recognised.. The model described by Bussey and Griffith (2017) acted as a background to support a change in practice, recognising the limited information available to support educators (Winstone et al., 2017a, b).

In the educational setting under review, at each clinical learning opportunity, oral feedback is paired with brief written feedback comments. In the current study, examining archival data, we focused on understanding the context and content of captured written feedback, rather than the verbal exchange which is not formally captured. This was a pragmatic decision but also recognised the persistent nature of the written feedback message which may be referred to later, where a verbal exchange cannot.

## Research aims

This study aimed to examine written feedback messages delivered by educators to learners during clinical learning sessions to determine whether a change in feedback ethos at a single dental school resulted in more effective feedback practice. In pursuit of this aim, the study examined differences between two time points, February 2017 and February 2019, before and after associated staff development interventions, to determine whether positive changes can be seen in the narrative feedback practice used, in relation to defined written feedback typologies associated with feedback roles described (Bussey & Griffiths, 2017). As a subsidiary aim, the research explored characteristics of written feedback messages, specifically concerning the valence and the length of feedback, in terms of feedback type as a means of furthering understanding of feedback practice.

Research questions:

Over the two-year period examined:

1. Did the type of written feedback change towards coaching?
2. How was emotion represented in terms of feedback type, and did emotional valence change?
3. How was the length of written feedback represented by feedback type, and did the number of words change?

## Methods

### Intervention

A programmatic assessment approach has been used by all educators in the school since 2017. This developmental approach aims to facilitate self-regulated development by providing learners with continuous low-stakes assessment and feedback, facilitated by an associated system, LiftUpp® (for reviews see Dawson, 2013; Dawson et al., 2015), which has been in use since 2012. However, some learners initially reported a feeling of over-assessment. To address this, the school commissioned a bespoke educator training day in July 2018 to encourage a coaching, or developmental, approach as a route to learner feedback acceptance and use. The applied training, facilitated by two academic psychologists, was attended by the vast majority of the educator team. The day aimed to instil a clear sense of the importance of good feedback processes and to instil confidence in their use. The objectives were to:

- Appreciate the crucial role of feedback in learner self-regulation;
- Understand the key psychological evidence around giving and receiving feedback;
- Recognise educator personalities and how to use them productively in providing meaningful *coaching* to students;
- Recognise the value of feedback provided to staff by students and how to use it positively to develop teaching;

These objectives were achieved through four facilitated interactive sessions. These focused on giving and receiving feedback; having difficult conversations; how to undertake coaching conversations; and supporting student adaptive self-regulatory behaviour. Before the session, all attendees completed a personality profile questionnaire, Quintax (Robertson et al., 1998). Confidential personality profile results were returned at the start of training and educators were given space, individually and during group discussion, to reflect how these understandings impact approaches to giving and receiving feedback.

Following this training, the school has continued to embed these understandings of the mechanisms of effective feedback practice, through ongoing departmental focus and individual staff training sessions. As an example, during these sessions, different feedback examples are discussed in terms of their impact on student acceptance and self-regulation. Finally, induction training also imbues new staff with an understanding of the importance of good feedback and what effective feedback delivery looks like. In pursuit of this new educators to the school spend two weeks shadowing experienced educators before independent practice. The approach described focused on first promoting the practice of feedback with a bespoke learning activity, moving to a point where practice is developed informally through continual discussion and refinement of feedback practice (Tripodi et al., 2020; Winstone & Boud, 2019).

### Handling archival feedback comment data

Written feedback data from two defined periods were extracted from LiftUpp®. These snapshots harvested all written clinical comments delivered during February 2017 (n = 1878) and February 2019 (n = 2294). These periods were selected as the former preceded staff development activities and the latter enabled consideration of a change in practice. Institutional ethics board approval, reference 6246, was granted for this archival analysis.

Written feedback comments are made following an assessment of each dental student’s performance in each clinical and clinical simulation session. Comments are recorded via an iPad interface to LiftUpp®. For added context, an average of 74 (range 68–77) and 72 (range 68–76) learners per year of study were registered in 2017 and 2019 respectively. Between the second and fifth year of study, clinical students experience between two and eight clinical sessions per week, depending on the year of study; with more senior learners experiencing a greater number of clinical sessions. All clinical educators (83 educators in 2017 and 66 educators in 2019) are expected to provide feedback in the manner described. Whilst these descriptive data are provided for additional context, they did not form part of the archival data extracted for analysis.

**Fig. 1:**
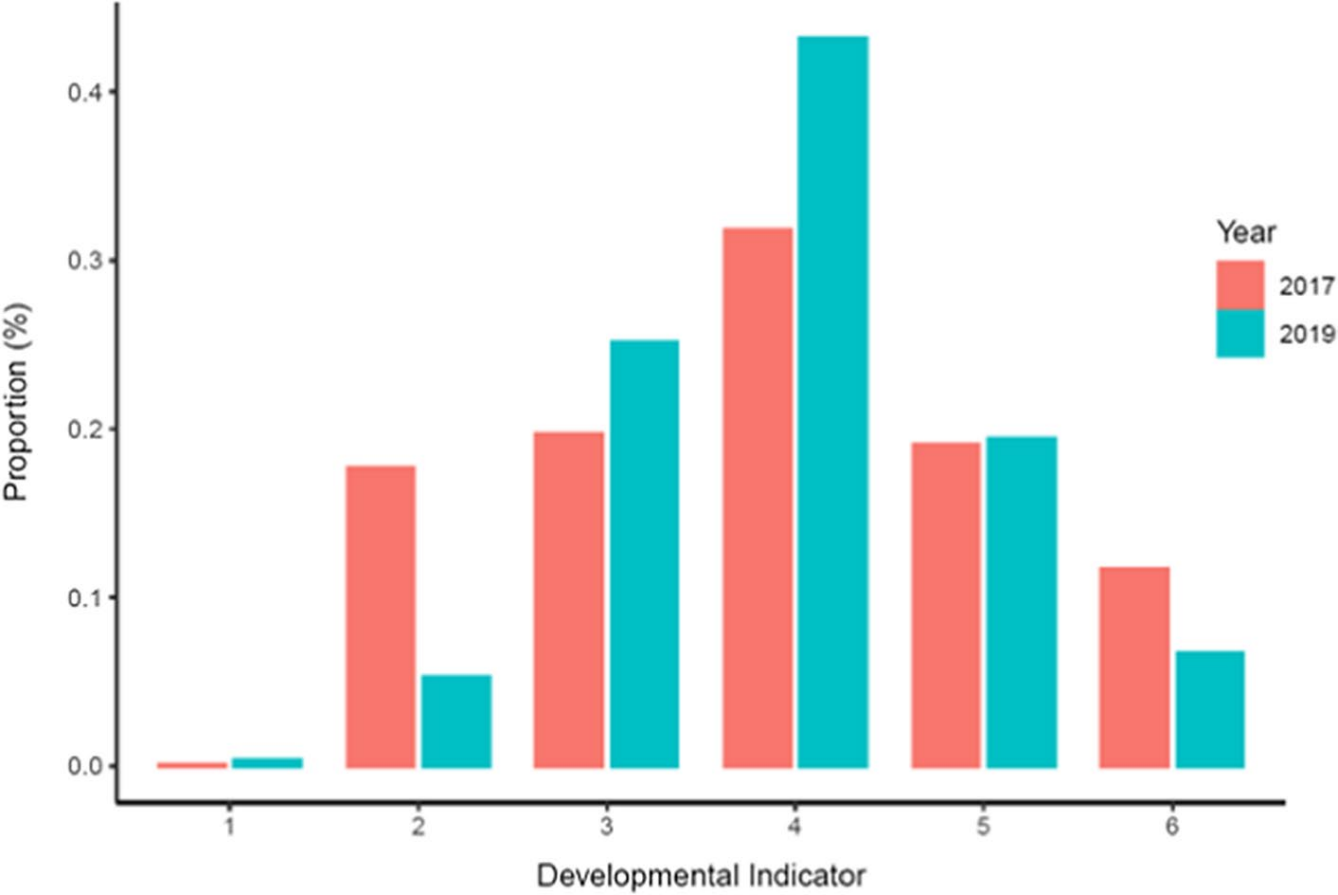
Proportions of Developmental Indicators split by Year

Educators also assess individual learner performance by applying developmental indicators (DI), using a 1–6 scale, to a broad range of assessment criteria, contextualised to each clinical and non-clinical activity (Oliehoek et al., 2017). Full independence is indicated by a DI of 6 where 1 indicates total dependence on an educator. An educator DI assessment is made for each contextualised criteria observed during a clinical session. Examples of these include professionalism, infection control, local anaesthesia, procedural knowledge and specific items related to technical procedures. On average, 11 domains per learner were assessed in each session in 2017 and 12 domains in 2019. For each criteria, if the educator assesses that a learner had not demonstrated independent practice, a DI of 4 or less, in any of the criteria, LiftUpp® requires a written feedback comment. If all aspects of the performance are assessed as independent, the need to record written feedback is at the educator’s discretion. At the end of each clinical observation, the learner and educator discuss feedback. Following this, the learner ‘signs-out’ on the iPad interface to confirm their awareness of feedback. Following this, learners can review feedback at any point via an online portal and request further opportunities for discussion with educators. Figure 1 shows differences in feedback DI assessments between the periods under review. Increased feedback DI assessments are seen on each side of the independence threshold with marked reductions in assessment towards the lower and highest end of the DI scale.

### Analytic approach

#### Categorisation of written feedback type

Three researchers, two with a background in clinical dentistry and a third researcher from a non-clinical, psychology background, examined the written feedback data independently. Each written feedback comment was classified as being predominantly one of three types; descriptive (administrator), evaluative (examiner) and coaching (mentor). These feedback types were used to describe written feedback made within each of the feedback roles described by Bussey and Griffiths (2017). Although some comments contained elements of more than one type such as evaluative and coaching, three main categories were retained to allow comparison with Bussey and Griffiths (2017). After the independent stages of classification, lack of concordance in the application of categories was resolved by negotiation across the rating team. Difficulties in applying classification often related to comments that were sentence fragments, recruiting assumptions about meaning. Other comments initially appeared to hold elements of two categories, and in a small number of cases three. Some agreement issues were resolved because they required clinical knowledge to classify appropriately. Although descriptive comments were more straightforward to identify, feedback was not classified as coaching unless it was felt that the learner would be able to take away information that would help them develop and improve. Any remaining comments were examined two, and sometimes three times until the research team arrived at a united position. Refined typologies with the supporting descriptors employed are identified in Table 1.

**Table 1:** Feedback comment typology descriptors and illustrative examples

Type (role)	Detailed descriptor	Examples of median comments made in 2017 and 2019
Descriptive (administrator)	Merely stated factual information about what happened during the clinical session. Impersonal in nature	Addition of denture tooth to existing partial upper denture [37, 2017] Also completed pre op assessment [93; 2019]
Evaluative (examiner)	Comments imply judgement and did not offer improvement advice. Comments could be appreciative or critical, or both. Largely impersonal	Optimal dental dam isolation [1105, 2017] Nice attempt, wisdom teeth are hard to reach [1302, 2019]
Coaching (mentor)	Every comment gave advice on how to improve performance, regardless of assessment element. Largely personalised within the learners context	Where you can't access teeth surface with ultrasonic, rely on your tactile sensation and use hand instruments. Also, calculus just below gingival margin on smooth surfaces lingually can easily be missed so pay attention to those areas. [1837, 2017] We discussed that positioning the film holder so that it is touching the arch that you are imaging before asking the patient to bite together helps to give a more reproducible position. Sometimes we need to tilt the film holder to account for the palate, when doing this, ask the patient to bring their bottom jaw forwards [2168, 2019]

Table 1 indicates written feedback comments within each written feedback type, illustrative comments represent those comments with median levels of emotional content. To further illustrate emotionality, the written feedback comments containing the greatest negative and positive valence respectively, were ‘Bite very shallow causing repetitive iatrogenic damage on the tissues’ [168, 2017], and ‘Excellent very neat prep’ [451, 2017], with the former assessed as descriptive and the latter evaluative written feedback types.

#### Quantitative analysis of feedback types and characteristics (emotional valence and word count)

In the first part of inferential analysis, the derived categorical written feedback data were examined in 3 × 2 Chi-square analysis to determine differences in the three feedback categories (descriptive, evaluative and coaching) by the two time periods (2017 and 2019). Next, feedback characteristics were examined. Specifically, the emotional valence of the language and the number of words used were examined. The sentimentR package (Rinker, 2019) was used to examine the emotional content of words and word combinations used in sentence-level statements, i.e. each written feedback comment at individual criteria level. The sentimentR package compares words against a predefined lexicon, NRC (Mohammad & Turney, 2013) which categorises 25,000 common words in the English language as to emotion type. The NRC lexicon is widely used within Natural Language Processing paradigms (Naldi, 2019; Silge & Robinson, 2017) and was originally funded by the National Research Council Canada (Mohammad & Turney, 2013). The emotional valence of language is returned as a score by comparing words to the NRC lexicon to arrive at an overall score for each statement (Silge & Robinson, 2017) and has been employed in understanding teachers use of emotions in written language (Chen et al., 2020). One advantage of sentimentR over other sentiment analysis approaches is that it accounts for valence shifters, such as ‘not’, which alter the meaning of the written word when taken in combination (Naldi, 2019). Finally, sentimentR also returns word counts for each comment from this analysis. After discovering that emotional valence and word count data were non-parametric, Kruskal–Wallis tests were conducted. Significant differences at the *p* < 0.05 alpha level were followed up with Dwass-Steel-Critchlow-Fligner (DSCF) pair-wise comparisons. Non-parametric tests were conducted using the jmv package (Selker et al., 2018). All analyses and visualisations were undertaken in R (Core Team, 2020).

## Results

Following the aims of the project, within and between two snapshots of archival data (2017 and 2019), the changes in the relative frequency of written feedback types used, their emotional valence and word counts were examined.

### Analysis of feedback frequency by feedback category and year

Overall, an increase in written feedback was observed between 2017 (n = 1878) and 2019 (n = 2294). Despite this increase, a reduction in the raw number of descriptive evaluations was seen between the two time points, where the evaluative and coaching categories saw an increase consistent with the overall increase in number. A 3 × 2 Chi-square test further examined observed differences in rating classification by year. This examination indicates an association between years and rating type $\chi^{2}$(2) = 61.09, *p* < 0.001. See Table 2 for a summary of observed and expected counts of feedback rating split by year. Figure S1 in supplementary information represents this graphically.

**Table 2:** Contingency table of observed versus expected ratings

Year	Descriptive	Evaluative	Coaching	Total
*2017*				
Observed	372	865	641	1878
Expected	285.39	880.93	711.68	1878
% within row	20%	46%	34%	100%
%within column	59%	44%	41%	45%
*2019*				
Observed	262	1092	940	2294
Expected	348.61	1076.07	869.32	2294
% within row	11%	48%	41%	1005
%within column	41%	56%	59%	55%
*Total*				
Observed	634	1957	1581	4172
Expected	634	1957	1581	4172
% within row	15%	47%	38%	100%
%within column	100%	100%	100%	100%

Given the data, as a proportion, a greater number of descriptive ratings were observed in the descriptive category than expected in 2017, indicated by positive standardised residuals, where fewer descriptive ratings are seen compared to expectation in 2019; indicating a significant reduction in descriptive evaluations between the two time points. In the evaluation category, neutral residuals are seen indicating consistency between observed and expected levels of evaluative ratings (regardless of an overall increase). Compared to expectation, fewer coaching comments were observed compared to expectation in 2017, indicated by negative standardised residuals, where positive residuals are seen in 2019 where the observed frequency exceed expectations, indicating a significant move towards coaching type feedback evaluations. Overall, marked differences are seen, with reduced descriptive evaluations and increased coaching feedback evaluations between the two time points; evaluative feedback rating proportions remain broadly consistent. See Table 2 for a comparison of observed written feedback category within the year under examination. For a graphical representation see Figure S2 in supplementary information.

In sum, data reported here indicates an overall increase in feedback between the two years. Despite this overall increase, a reduction in descriptive feedback was observed compared to expectation. Whilst evaluative ratings remained in line with expectation, an increase in coaching feedback, above expectation, was observed.

### Analysis of feedback sentiment split by rating and year

The emotional valence of written feedback data is described in Table 3 split by rating type and year; a summary of median written feedback comment by category is also shown in Table 1. As data are non-parametric, data is presented in terms of medians, and minimum and maximum statistics. Feedback sentiment scores range in the data from − 1.22 to + 1.62, representing the most negative and most positive feedback comments. A neutral comment, with neither positive nor negative sentiment would be recorded as 0, this can be seen in relation to descriptive feedback comments in each of the years under examination. These data are presented graphically in the supplementary information at Figure S3.

**Table 3:** Descriptive analysis of feedback sentiment data

Year	Descriptive	Evaluative	Coaching	Total
2017	0.00 (− 0.72–0.76)	0.25 (− 1.22–1.62)	0.10 (− 0.72–1.44)	0.11 (− 1.22–1.62)
2019	0.00 (− 0.57–1.48)	0.35 (− 0.53–1.61)	0.11 (− 0.95–1.13)	0.18 (− 0.95–1.61)
Total	0.00 (− 0.72–1.48)	0.31 (− 1.22–1.62)	0.11 (− 0.95–1.44)	0.15 (− 1.22–1.62)

Two Kruskal–Wallis tests were used to explore differences in average sentiment score, firstly by year and then by feedback rating. Average feedback sentiment differed by year $\chi^{2}$ (1) = 25.80, *p* < 0.001, *ε*^*2*^ = 0.006 indicating a small effect, and betraying the overall increase between the two time-points.

The second Kruskal–Wallis test examined average sentiment in feedback split by feedback category, indicating significant differences overall $\chi^{2}$(2) = 380.10, *p* < 0.001, *ε*^*2*^ = 0.006. DSCF pairwise comparisons to examine differences feedback rating category in both years. Lower average sentiment was seen in descriptive ratings when compared to evaluative ratings (*W* = 22.33, *p* < 0.001); evaluative ratings reported higher average sentiment than coaching ratings (*W* = − 21.94, *p* < 0.001); and coaching ratings reported significantly higher median ratings than descriptive ratings (*W* = 9.19, *p* < 0.001).

To further explore the sentiment data differences in each feedback rating category split by year was examined. Mann–Whitney U tests were used to examine the three rating types. Only evaluative ratings exhibited differences in feedback rating between 2017 and 2019, *U* = 404,984, *p* < 0.001, *d* = 0.006, a small effect size. No differences were seen in descriptive feedback ratings, *U* = 47,394, *p* = 0.543 or coaching feedback ratings, *U* = 295,836, *p* = 0.542. See Table 3 for descriptive statistics. Figure S4 in supplementary information displays these results graphically.

To summarise, in both years descriptive feedback is largely neutral and does not differ between years. Evaluative feedback holds the most positive valence, with overall valence increasing between years. The emotional valence of coaching feedback does not differ between years, however, is significantly lower in valence than evaluative comments and significantly higher in valence terms than descriptive ratings.

### Analysis of feedback comment word count split by category and year

To further investigate feedback message characteristics, the number of words were also examined as a function of both the year and feedback-rating category, see Table 4 for a descriptive summary.

**Table 4:** Median, minimum and maximum word counts split by feedback category and year

Year	Descriptive	Evaluative	Coaching	Total
2017	5 (1–80)	6 (1–88)	14 (3–127)	8 (1–127)
2019	7 (1–57)	8 (1–133)	19 (3–110)	12 (1–133)
Total	6 (1–80)	1 (7–133)	17 (3–127)	10 (1–133)

A first analysis, using a Kruskal–Wallis test, reported that significantly more words were used by educators in 2019 than in 2017, $\chi^{2}$ (1) = 162.85, *p* < 0.001, *ε*^*2*^ = 0.04, indicating a small effect. Next, differences were seen in the number of words used by educators as a function of the rating category, $\chi^{2}$(2) = 1340.76, *p* < 0.001, *ε*^*2*^ = 0.324, indicating a large effect. DSCF pairwise comparisons revealed that more words were used in evaluative feedback ratings than descriptive feedback ratings (*W* = 9.61, *p* < 0.001). In addition, more words were used in coaching feedback by educators than both descriptive (*W* = 37.76, *p* < 0.001) and evaluative feedback (*W* = 47.00, *p* < 0.001).

To follow this analysis up, using Mann–Whitney comparisons, significant differences were seen in the number of words used by educators between 2017 and 2019 in the descriptive category (*U* = 42,148.50, *p* < 0.001); also in the evaluative category (*U* = 383,096.00, *p* < 0.001); and finally in the coaching category (*U* = 210,546.00, *p* < 0.001). See Table 4 for descriptive statistics. Please refer to Figure S5 in supplementary information for a graphical summary of these data.

To summarise, there was a consistent and significant increase in the number of words used by educators, overall and between the two snapshots. Descriptive feedback employed the fewest words, evaluative feedback was next with coaching feedback using the most words. A large increase in words was seen in 2019 from 2017 in the coaching category, with smaller but still significant increases seen in evaluative and descriptive rating categories.

## Discussion

Changes seen after school-wide effort to promote more effective feedback included a reduction in descriptive written feedback, an increase in coaching feedback with little change in evaluative feedback. An increase in positive emotional valence was seen in evaluative feedback only, and an increase in the number of words written across all types of written feedback. Coaching feedback contained the most words, and descriptive comments the fewest.

When comparing the findings of the current study to Bussey and Griffiths’ (2017), lower proportions of descriptive, and higher proportions of both evaluative and coaching written feedback comments are seen, in both 2017 and 2019. Several plausible explanations include different feedback transmission approaches, variance in feedback practice by institution, clinical domain and academic level.. To illustrate, the current study examined written feedback provided to undergraduate preregistration dental students, where Bussey and Griffiths’ examined feedback provided to qualified doctors undergoing postgraduate training. The coaching approach increased between the timepoints in the current study, perhaps indicating greater use of feedback approaches that combine assessment of, and for, learning than had been seen previously in the current institution. Understanding the student perspective is an important part of understanding feedback practice. We tentatively note that the product of the effort described in the current study is that the school currently ranks highest within its institution and exceeds the mean of the UK dentistry sector by some 20.8%, in the Assessment and Feedback domain in the latest National Student Survey.

These encouraging results lend weight in clinical education to the pairing of both formal and informal learning and development opportunities which support coaching focused feedback practice that enocourage reflection We contend that a focus on coaching in feedback practice, such as that seen here is also increasingly mindful of the learner perspectives and the complex interplay of personality as part of institutional processes and supports dialogues that may encourage feedback uptake (Freeman et al., 2020; Winstone et al., 2017a, b).These coaching dialogues move away from more traditional, educator centric, feedback practices that include vague, descriptive or evaluative feedback that is generally known to have limited usefulness (Bösner et al., 2017; Shaughness et al., 2017). Often technology-mediated methods of feedback delivery can encourage a one-way, transmission approach where learners focus on grade rather than feedback and understanding (Carless & Boud, 2018; Winstone et al., 2020). The practice examined in the current study recognises that written feedback comments delivered by the learning management system are part of a wider approach to feedback. Brief written feedback, removed from the dialogic context may, overall, be different in reality (Shaughness et al., 2017)., However, learners may review this feedback much later with a different lens. At that point, written feedback may be the only data learners have access to and may, as a result, be their sole source of improvement data. It may be then that coaching type written feedback comments are a crucial part of the dialogue.

It was interesting to note that coaching feedback in the current study was more neutral in tone when compared to evaluative feedback, which tended towards positive sentiment. Evaluative feedback also increased to become more positive between the two time periods. In part, this could be attributed to discussion during educator training, which resulted from previously unpublished research, which asked educators to consider the impact of emotions in written feedback on learner motivation. However, the emotional valence of feedback is known to have differential impacts on learner feedback uptake (Pitt et al., 2020). Feedback that sandwiches a developmental message within positive sentiment, may help dampen learner and educator emotions in difficult interactions, however, can a deleterious effect on feedback uptake (Molloy et al., 2020). No strong inferences are made here about the emotional valence of feedback, however, we note the neutral characteristics associated with coaching feedback. If coaching feedback supports learners towards feedforward, this may support learner towards greater independence and the positive upward learning spiral suggested in self-regulated learning theory (Hattie & Timperley, 2007; Zimmerman, 2000).

As with any research, these findings are limited. This study only examined written feedback in one dental school, it is unclear how these findings would apply in other educational settings. From research, we are aware that similar settings, using similar approaches, experience similar challenges (see, for example, Freeman et al., 2020). However application may differ across settings, as exemplified by Bussey and Griffiths (2017) and further research continues to be warranted in this area. The classification of written feedback to three categories in the absence of other contextual data also presented a challenge and may limit these findings. However, the research team aimed to reach coding agreement through discussion. Some feedback could easily be allocated to a category, however, other comments were less straightforward. Written feedback that most challenged the research team were those narratives that could perhaps have been either evaluative or coaching. We took the view that where a comment supported a learner in understanding where they had to go next most clearly represented coaching feedback, representing the idea of feedforward (Hattie & Timperley, 2007). The absence of context, including the broad situational factors and the associated developmental indicators, was a final limitation of examining written feedback. These factors may speak to complexity not captured in the current study. In particular, the categorical inferences attached by the research team to written feedback may not have the same meaning that exists within an in-person feedback dialogue which would incorporate cues from language, relationships, and developmental indicators that might have led learners to greater acceptance of feedback and action (Jellicoe & Forsythe, 2019; Winstone et al., 2017a, b). From this study, it is not clear how much verbal feedback is provided in support of written feedback. Unpublished evidence from the first author indicates that educators take the time to provide verbal coaching, however, this is not currently recorded. As a result, further work will be undertaken to examine the learner perspective on written feedback. A further line of enquiry will also examine how learners perceive the discussions and relationships that are associated with feedback in each of the categories discussed here, and those which support feedback uptake.

To conclude, we noted that written educator feedback narratives, moved toward an emotionally neutral coaching approach, signalling recommendations for next steps, whilst remaining relatively concise. There was also a move away from short feedback narratives that were both descriptive and neutral, and evaluative feedback comments that tended towards greater positive emotion. We add to knowledge here by demonstrating that a focus on feedback practice that pair formal and informal educator learning opportunities can have a positive impact on practice by moving the feedback conversation towards learner-centred coaching dialogues. This continuing journey in our current institution seeks to understand both educator and learner response. We recommend further research to explore learner reaction to supportive feedback dialogues in clinical education.

## Supplementary Information

Below is the link to the electronic supplementary material.

Supplementary file1 (DOCX 265 KB)
