# Supplementary material for: How does a move towards a coaching approach impact the delivery of written feedback in undergraduate clinical education?
Source: Adv Health Sci Educ Theory Pract. 2021 Sep 14;27(1):7–21. doi: 10.1007/s10459-021-10066-7 (PMC8938375; doi:10.1007/s10459-021-10066-7)

Supplementary information

Figure S1: Clustered histogram indicating the count of feedback rating categories split by year


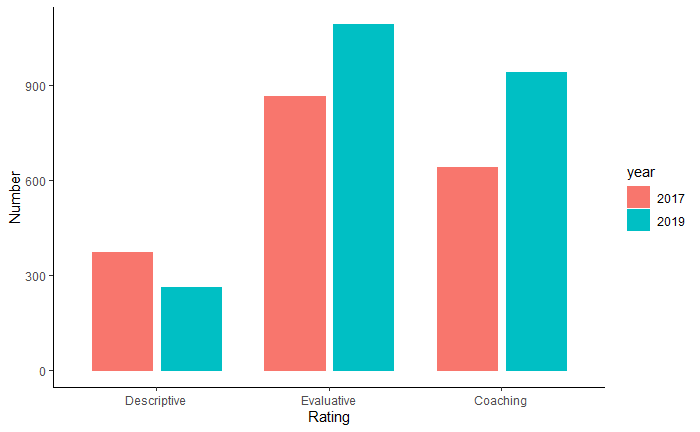


Figure S2: Chi-square contingency table (standardised residuals visualised)


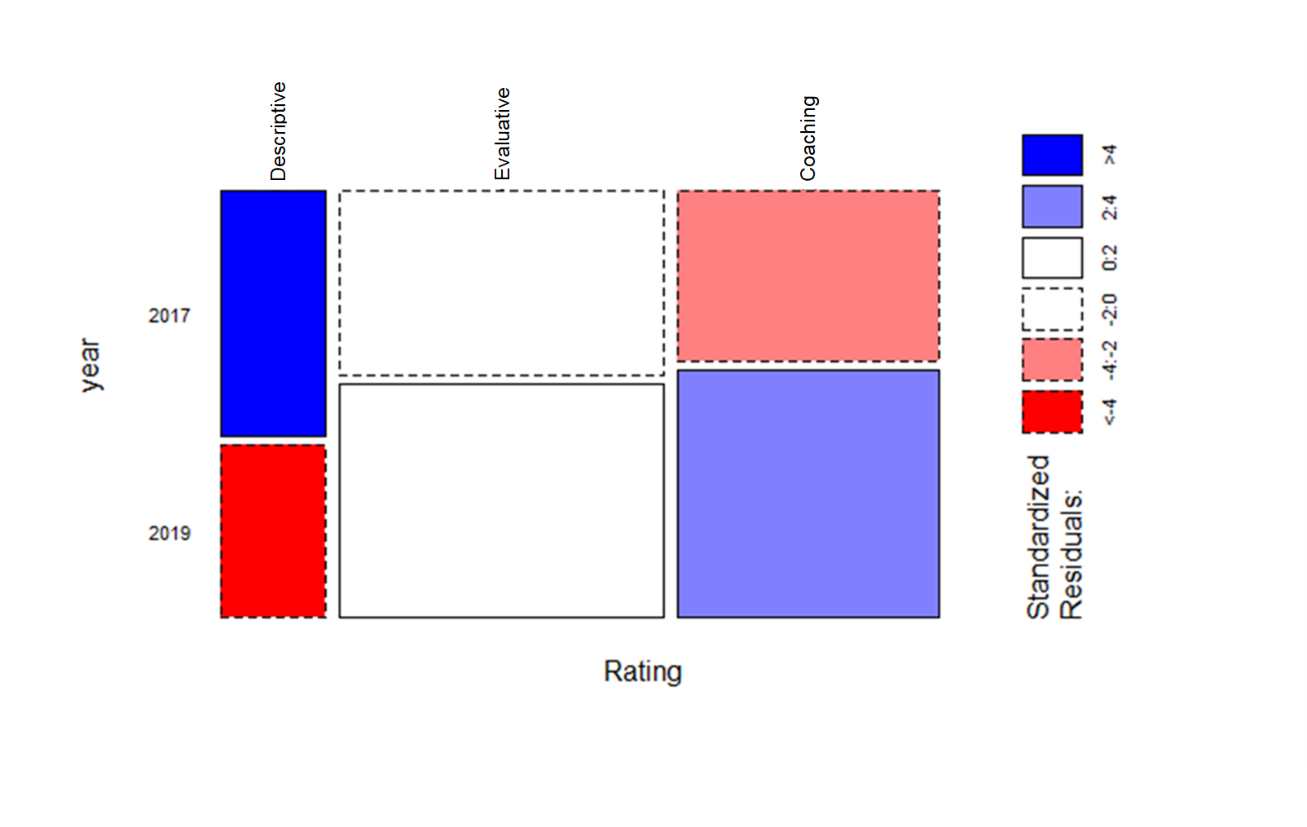


Figure S3: Nested histogram of feedback sentiment split by feedback category (dashed lines represent median values)


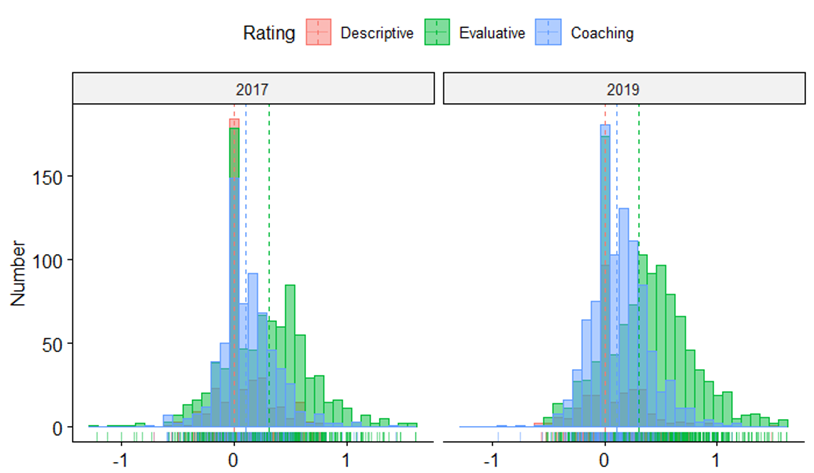


Sentiment Score

Figure S4: Error bar plot of average feedback sentiment split by feedback category and year. Error bars indicate Mean ratings and 95%CI for each rating split by year; non-central observations to the error bar indicate median values.


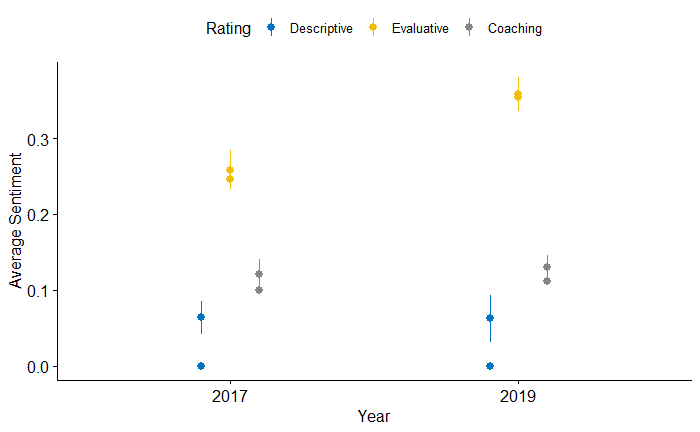


Figure S5: Error bar plot of feedback word count split by feedback category and year. Error bars indicate Mean ratings and 95%CI for each rating split by year; non-central observations to the error bar indicate median values.


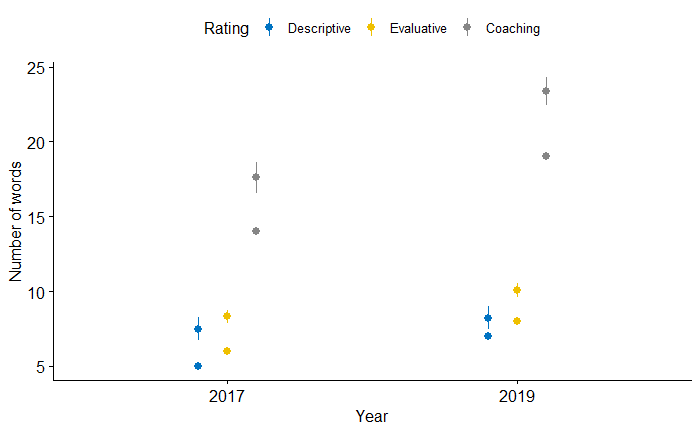

Supplement: Supplementary file 1 — Supplementary file1 (DOCX 265 KB) [file 10459_2021_10066_MOESM1_ESM.docx]
